# ^11^C–acetate PET/CT: a potential alternative of transcranial biopsy for grading cerebral gliomas

**DOI:** 10.1007/s00259-018-3950-2

**Published:** 2018-02-19

**Authors:** Jun Hatazawa

**Affiliations:** Department of Nuclear Medicine and Tracer Kinetics, Osaka University Graduate School of Medicine, 2-2 Yamadaoka, Suita, Osaka 565-0871 Japan

Cerebral gliomas are one of the most intractable diseases. Therapeutic strategy has been determined according to World Health Organization (WHO) grading based on the histopathological findings. Biopsy is chosen to categorize brain tumors from grade I to IV of WHO grading. Different from other organs, biopsy of brain tumor is more invasive because of transcranial sampling. In addition, heterogeneous features of brain tumors disturb accurate diagnosis because of limited sampling points and sometimes lead to under-grading of the tumor.

In the current study, Kim and colleagues employed ^11^C–acetate, a metabolic biomarker of glial cells, of which the uptake is mediated by mono-carboxylate transporter expressed specifically on the glial cell membrane [1]. The study demonstrated that the magnitude of ^11^C–acetate uptake was significantly different among WHO grading, where more uptake was associated with higher grade. The study also demonstrated that ^11^C–acetate PET/CT parameters such as tumor to choroidal plexus count ratio and metabolic tumor volume are independent predictors of progression-free survival and overall survival by multivariate analysis. Although histopathological WHO grading and gene abnormality such as IDH 1 mutation, MGMT methylation, and 1p19q co-deletion studied by biopsied tissue samples were predictors of survival in the univariate analysis, these were not significant after removal of confounding factors by multivariate analysis.

In clinical practice, a diagnosis of non-enhancing brain tumor by CT and MRI is most difficult because 36% of non-enhancing tumors were of high grade [2]. It was also indicated that 13% of enhancing tumors were low grade. The diagnostic ability of CT and MR for brain tumor grading based on the use of contrast medium has limitations. In the current study, significant difference in ^11^C–acetate accumulation between grade II and grade III gliomas suggested that non-enhancing high-grade brain tumors (mostly grade III) can be distinguished from non-enhancing low-grade tumors by ^11^C–acetate PET/CT. Figure 2c and 2d in the article by Kim et al. demonstrate such a case where ^11^C–acetate accumulated to non- or little-enhancing lesion of anaplastic oligoastrocytoma (WHO grade III).

MR imaging is now the standard imaging procedure for brain tumors. ^11^C–acetate PET/CT is a non-invasive alternative of transcranial biopsy for grading gliomas. It provides an independent predictor of a patient’s prognosis even better than histopathological findings and gene abnormalities. Development of cell population-specific probes further facilitates a use of PET/CT in the management of patients with cerebral gliomas.
